# Modeling Brain Resonance Phenomena Using a Neural Mass Model

**DOI:** 10.1371/journal.pcbi.1002298

**Published:** 2011-12-22

**Authors:** Andreas Spiegler, Thomas R. Knösche, Karin Schwab, Jens Haueisen, Fatihcan M. Atay

**Affiliations:** 1 Max Planck Institute for Human Cognitive and Brain Sciences, Leipzig, Germany; 2 Institute for Biomedical Engineering and Informatics, Technical University Ilmenau, Ilmenau, Germany; 3 Bernstein Group for Computational Neuroscience Jena, Institute of Medical Statistics, Computer Sciences and Documentation, Jena University Hospital, Friedrich Schiller University Jena, Jena, Germany; 4 Max Planck Institute for Mathematics in the Sciences, Leipzig, Germany; Indiana University, United States of America

## Abstract

Stimulation with rhythmic light flicker (photic driving) plays an important role in the diagnosis of schizophrenia, mood disorder, migraine, and epilepsy. In particular, the adjustment of spontaneous brain rhythms to the stimulus frequency (entrainment) is used to assess the functional flexibility of the brain. We aim to gain deeper understanding of the mechanisms underlying this technique and to predict the effects of stimulus frequency and intensity. For this purpose, a modified Jansen and Rit neural mass model (NMM) of a cortical circuit is used. This mean field model has been designed to strike a balance between mathematical simplicity and biological plausibility. We reproduced the entrainment phenomenon observed in EEG during a photic driving experiment. More generally, we demonstrate that such a single area model can already yield very complex dynamics, including chaos, for biologically plausible parameter ranges. We chart the entire parameter space by means of characteristic Lyapunov spectra and Kaplan-Yorke dimension as well as time series and power spectra. Rhythmic and chaotic brain states were found virtually next to each other, such that small parameter changes can give rise to switching from one to another. Strikingly, this characteristic pattern of unpredictability generated by the model was matched to the experimental data with reasonable accuracy. These findings confirm that the NMM is a useful model of brain dynamics during photic driving. In this context, it can be used to study the mechanisms of, for example, perception and epileptic seizure generation. In particular, it enabled us to make predictions regarding the stimulus amplitude in further experiments for improving the entrainment effect.

## Author Summary

Neuroscience aims to understand the enormously complex function of the normal and diseased brain. This, in turn, is the key to explaining human behavior and to developing novel diagnostic and therapeutic procedures. We develop and use models of mean activity in a single brain area, which provide a balance between tractability and plausibility. We use such a model to explain the resonance phenomenon in a photic driving experiment, which is routinely applied in the diagnosis of various diseases including epilepsy, migraine, schizophrenia and depression. Based on the model, we make predictions on the outcome of similar resonance experiments with periodic stimulation of the patients or participants. Our results are important for researchers and clinicians analyzing brain or behavioral data following periodic input.

## Introduction

Electrophysiological measurements such as *magneto-* and *electroencephalography* (M/EEG), *local field potentials* (LFP) or single unit recordings contain rich information on brain function, which may be related to specific cognitive processes, to general brain states, or to certain pathological conditions. For example, it is known that stimulation by repetitive light flashes entrains the intrinsic alpha EEG rhythm (i.e., frequency entrainment). Neurons in the human visual cortex synchronize their firing to the frequency of flickering light (at rates of about 5 to 30 Hz), causing the EEG alpha frequency to change toward the stimulation frequency [1], [2], [3]. Clinically, this resonance effect is called photic driving. The effect of photic stimulation of the human EEG was first studied in the 1930s and 40s [4]. As early as 1947, photic driving was reported in three cases as a potential cause for epileptic activity in patients [5]. A review of the clinical routine can be found in Niedermeyer et al. [4]. The occurrence of this effect is often interpreted as an indicator for the functional flexibility of the cortex and thus as a sign of healthiness. Today, photic driving is widely used as an activation method in clinical practice, for instance, in epilepsy, migraine, schizophrenia or depression [6], [7], [8]. Note, however, that only 50 to 80% of healthy volunteers show a response in the alpha range of EEG [9], .

Basic properties of the alpha rhythm during photic driving have been investigated by electroencephalographic methods [11], [12], [13], [14]. A closer examination of electroencephalographic photic driving effects was given by Herrmann [2]. In that investigation, a flicker stimulus from 1 to 100 Hz in 1-Hz steps was presented. Miranda de Sa and Infantosi [10] stimulated at 4, 5, 6, 8, 10, and 12 Hz and showed that stimulation close to the alpha peak was much more effective. The quantification of photic driving from EEG as well as MEG recordings was carried out for the first time by Kalitzin and Parra [15], [16]. They estimated the phase clustering index of harmonically related frequency components in the EEG and MEG of normal controls and epileptic patients during light stimulations with 10, 15 and 20 Hz. Topographic effects of encephalographic photic driving in the case of children and adolescents were described by Lazarev et al. [9], [17], for patients with migraine by de Tommaso et al.[18], and for patients with schizophrenia by Jin et al. [19].

In order to gain further insight into mechanisms underlying such brain resonance effects and their relevance to brain function and pathology, as well as to make predictions concerning the stimulation parameters, generative models can be used. Such models are called biologically plausible if their state variables and parameters are biophysically meaningful. By fitting the model parameters to measurements, one can test hypotheses on the implementation of brain function. To ensure that this inversion is mathematically tractable and at the same time physically meaningful, the model must strike a balance between mathematical simplicity and biological realism. One class of models designed to meet these criteria is referred to as *neural mass models* (NMMs) (e.g., [20], [21], [22], [23], [24], [25], [26]). NMMs describe neural function at a mesoscopic level [27], [28], in contrast to single neuron models such as simple integrate-and-fire models [29] and the more elaborate Hodgkin and Huxley type of models (e.g., [30], [31]). NMMs quantify the mean firing rates and mean postsynaptic potentials (PSPs) of neuronal populations, the *neural masses* (NMs). Although, at the microscopic level, single neurons are considered the primary computational units of the brain's architecture [32], [33], it is also widely accepted that relevant information processing underlying brain function in both healthy and diseased states can be carried out by ensembles of interacting neurons at the mesoscopic level (e.g., [27], [28], [34], [35], [36], [37], [38]). In other words, NMMs describe brain activity on a scale that is highly relevant to brain function [39], [40], [41]. Moreover, when EEG or MEG data are used, NMMs have also the advantage that they predict exactly what is measured by these modalities, namely coherent activity in entire populations of neurons.

However, this type of modeling also involves a number of simplifications that may lead to limitations. First of all, it is based on a simplified notion of the function of a neuron, namely the firing rate model: The neuron convolves the rate of incoming spikes with an alpha-shaped function and thereby generates a change in membrane potential (PSP), and produces an output spike rate that is a non-linear (e.g., sigmoid) function of the membrane potential. These are the most important aspects of neuronal function. However, in the brain, things are usually more complicated. For examples, , modeling is made difficult due to feedback influence of action potentials on the dendritic membrane potentials (back propagation) [42], specific intrinsic firing patterns (e.g., bursting) [43] and dendritic hierarchies [44]. It remains to be investigated if and to what extent such physiological details affect the properties of NMs at the mesoscopic level. A second simplification that leads to limitations is that spike time dependent effects will be missed since the model relies on firing rates rather than on actual spikes. Third, as the distributions of the neural states are simply described by their means, the impact of higher statistical moments is ignored. In order to capture the variability within a NM, one may use the Fokker-Planck formalism [45], [46], [47]. Finally, NMMs approximate the spatial scale of neuronal populations to be point-like [22], [23], [24], [28], ignoring the domain of spatial dynamics. In that line, the approach can be generalized, leading to neural field models [20], [25], [26], [27], [28], [48], [49], which take into account the spatial extent of neural circuitry by dealing with aggregated activities in the vicinity of a given location. This puts field theories somewhere between neural mass theories and discrete neuronal networks, allowing them to address, for instance, distance-dependent delays. A quantitative analysis of neural field models can be found in Atay and Hutt [50], [51], for example.

In this work, we use a particular local network of NMs first described by Jansen and Rit [23], [52], based on earlier works of Lopes da Silva et al. [24], [53] and Zetterberg et al. [54]. This NMM comprises an elementary circuit of three interconnected NMs (i.e., pyramidal cells and excitatory and inhibitory interneurons) meant to account for a cortical area, such as the primary visual cortex in our photic driving experiment. Although local neuronal circuits can be very complex [55] and may be modeled using more than three NMs (e.g., [56]), the circuit used here is the most reduced representation of the features that are relevant for the temporal dynamics, that is, positive and negative feedback loops. The Jansen and Rit structure has been shown to account for both oscillatory [57] and seizure-like EEG recordings [58], [59]. Its dynamic behavior, in terms of stabilities and bifurcations, was first characterized by Grimbert and Faugeras [60] and, more generally, by Touboul [61] and Spiegler et al. [62]. Several such NMMs can be combined to describe networks of coupled cortical areas and account for more complex transient and oscillatory behaviors [23], [57], [59], [63], [64], [65]. The Bayesian inversion of such network NMMs given M/EEG data (referred to as *dynamic causal modeling* (DCM) [64], [66]) has been successfully used for the analysis of event-related [64], [67], [68] and steady-state responses [69].

To date, the dynamics of this system has been systematically investigated only under the assumption of constant extrinsic input levels, thereby allowing the system to settle in a stable state (e.g., fixed point or limit cycle) [60], [61], [62]. However, in a photic driving experiment, one has to consider rhythmic input. Moreover, the model's response to such input is also of great importance in many other settings, since, in the brain, such local neural circuits are embedded in global brain networks and may experience high amplitude time-varying input from other parts of the brain. Because neuronal ensembles tend to oscillate intrinsically, such input is very often periodic, as evidenced by the widespread occurrence of rhythmic activity in both extracranial and intracranial recordings [70].

In this paper, we use a continuous-time periodic function as model input approximating a periodic train of pulses. In this continuous function, each single pulse is similar (but not equal) to the single event used by Jansen and Rit for eliciting visual evoked potentials [23], [52], or used in dynamic causal modeling (e.g., [63], [66]). We systematically vary both amplitude (intensity) and frequency of the stimulation within the effective ranges provided by Spiegler et al. [62]. We find the frequency entrainment effect spreading over broader stimulus frequencies for higher stimulus intensities, while away from the entrainment ranges, we find complex behavior, including periodic, quasi-periodic, and chaotic dynamics. The latter behavior, in particular, provides continuous spectra. Networks of such chaotic NMMs (incorporating network variability, for example, by different characteristic constants of time and potential) can be used to describe colored noise sources that produce continuous portions in the spectra, such as 1/*f*-characteristics, that are commonly observed in M/EEG or LFP data [71]. Finally, we fit the output of the periodically forced NMM to data from the photic driving experiment in terms of the largest Lyapunov exponent and frequency detuning. The largest Lyapunov exponent measures the exponential separation or convergence of nearby trajectories. It thereby quantifies the predictability or, at the other extreme, the chaoticity of the behavior of the system and has been demonstrated to be an important marker for pathologically altered brain dynamics, especially in epilepsy [72], [73], [74]. In this way, we show that the NMM is a suitable model for the dynamics of brain resonance phenomena at the cortical level and demonstrate that useful predictions concerning the parameter choice of entrainment experiments can be derived.

To our knowledge, this is the first study to investigate a photic driving experiment using a NMM. We demonstrate that with this NMM, one can explain effects of complex behavior in such an experiment. The results also indicate that a relatively simple model of a local neural circuit is capable of producing surprisingly complex and diverse phenomena, which are observable in brain data and relevant to the explanation of brain function.

## Results

In our previous work on the extended Jansen and Rit neural mass model (NMM) for a cortical area [62], we found a self-sustained oscillation due to a stable limit cycle with a certain intrinsic frequency for constant input. Forcing such a limit cycle with periodic input to the NMM causes accelerations and/or decelerations of the oscillation, depending on the timing. If their cumulative effect is non-zero, entrainment occurs. For more details on the precise mechanism of entrainment effects, see [75], [76].

**Figure 1:**
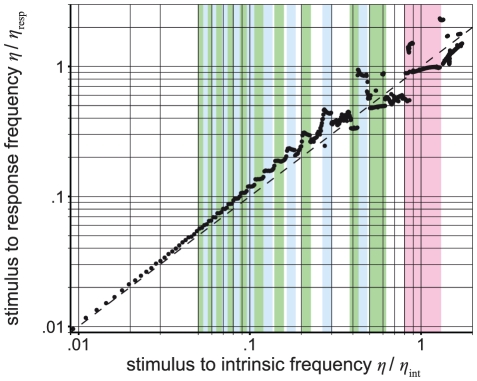
Frequency entrainment effects in a periodically forced neural mass model of a cortical area. A frequency-detuning curve refers to the ratio of stimulus to characteristic mean response frequency plotted against the ratio of stimulus to intrinsic frequency, for the normalized stimulus amplitude of 1.5. The entrainment ranges around the intrinsic frequency and its subharmonics are shown in red and in blue/green. Such an entrainment around the intrinsic frequency can be found in photic driving experiments (e.g., [81]).

**Figure 2:**
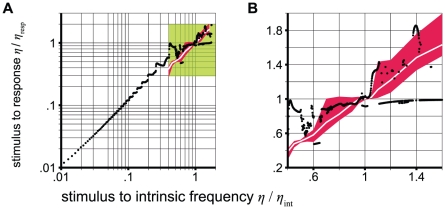
Entrainment effect found in the experimental data and model. For the experiment, the mean over subjects is shown as white lines and the region between the 5% and 95% quantiles is covered by red areas. For the model (black dots), the amplitude configuration that best fits the largest Lyapunov exponents of the experiments is used (see Comparison section in Materials and Methods). The entrainment effect is shown for **A** the stimulus frequency range of the model and **B** the stimulus range used in the experiments (green area in A).

Indeed, we observe frequency entrainment, that is, the cortical area responds with the stimulus frequency instead of the intrinsic frequency, thus forming a plateau in the frequency-detuning curves (see colored ranges in Figure 1). The detuning curve shows intrinsic behavior that is characterized by a typical repetitive s-shape. With increasing frequency, this s-shape becomes more pronounced. For the model with the stimulus amplitude that fits the experimental data best, this s-shape pattern of the detuning-curve is frequently interrupted near the stimulus hitting the intrinsic frequency 0.5≤*η*/*η*_int_≤1.5 by complex behavior such as chaos (see below, as well as Figure 1 and Figure 2). Apart from the interruption of the repetitive s-shape pattern by irregularities around the intrinsic frequency *η*_int_, the general trend of frequency detuning seems to be shifted towards the intrinsic frequency *η*_int_ by the response frequencies *η*_resp_, which explains the experimental data (see B in Figure 2). Moreover, stimulating near the intrinsic frequency the response matches the stimulation frequency and entrainment occurs (see Figure 1 and Figure 2).

**Figure 3:**
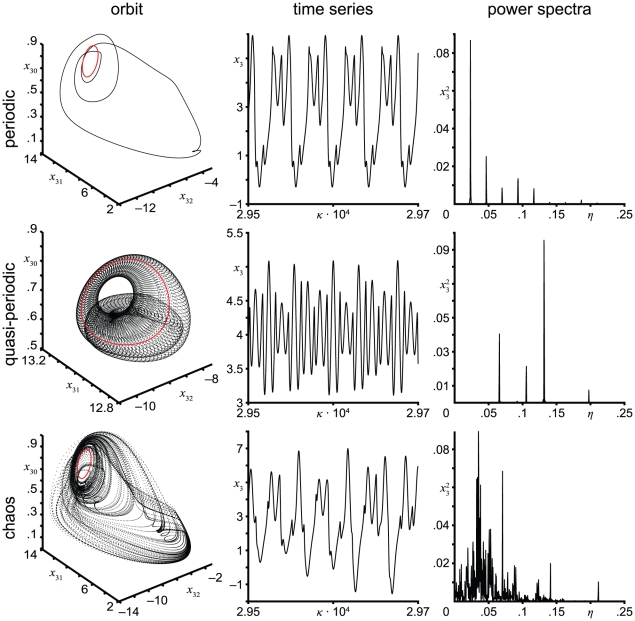
Complex behavior occurring in the periodically forced neural mass model of a single area. Orbits, time series, and power spectra (columns) are shown for three configurations (rows) displaying (top-down) periodic (normalized input amplitude; normalized input frequency: 3.6301; 9.33·10^−2^), quasi-periodic (1.5; 7.59·10^−2^) and chaotic behavior (3.6301; 7.05·10^−2^). The orbits are in the state space of normalized postsynaptic potentials of pyramidal cells caused by both interneurons (*x*_30_) as well as at both excitatory and inhibitory interneurons caused by pyramidal cells (*x*_31_ and *x*_32_). The red circle represents the stable limit cycle (i.e., harmonic oscillation) arising from Andronov-Hopf bifurcations performed by the unperturbed system. The time series and the power spectra are shown for the normalized postsynaptic potentials of pyramidal cells (which are related to M/EEG). Periodic behavior is characterized by a closed orbit (limit cycle) and a discrete power spectrum with peaks at commensurable frequencies. Quasi-periodic behavior is characterized by trajectories forming an invariant *n*-dimensional torus and discrete power spectra with peaks at incommensurable frequencies. Chaotic behavior is indicated by a strange attractor, that is, a bounded attracting set in which all trajectories are unstable and nearby trajectories diverge locally from each other exponentially, and a broadband power spectrum.

**Figure 4:**
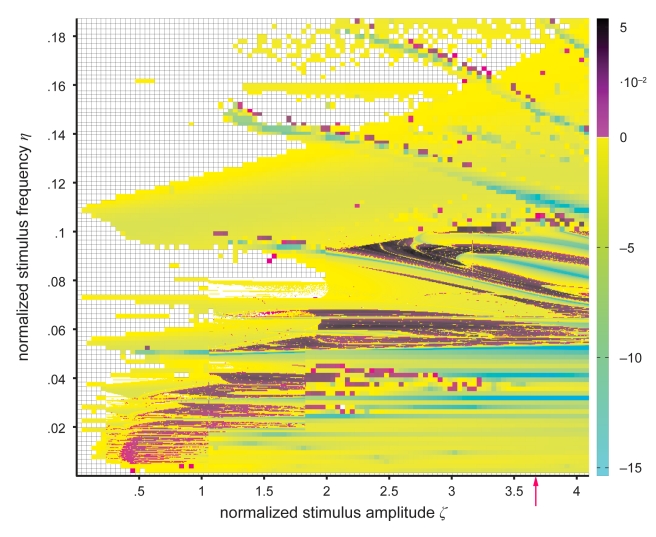
Largest Lyapunov exponent in parameter space. The map shows the largest Lyapunov exponent *λ*_1_ as a function of stimulus amplitude and frequency, indicating the sensitivity of the periodically forced neural mass model to initial conditions. Positive exponents (magenta to black) reflect diverging trajectories irrespective of how close they are, and thus chaos in the system. They also measure the entropy of an attracting set for all cases because the maximum number of positive Lyapunov exponents for any parameter configuration is one. Zero exponents (white) indicate neutral stability, and negative exponents (cyan to yellow) reflect frequency locking. Arnol'd tongue structures (i.e., resonance zones) are indicated by negative largest Lyapunov exponents due to the phase locking between system kinetics and the stimulus. The red arrow indicates the amplitude for which the experimental data fits best (see Figure 8 and Comparison section in Materials and Methods). Several parameter regions with scattered, presumably fractal patterns of chaotic regime are shown at a finer resolution of normalized stimulus amplitude and frequency.

Outside the entrainment ranges, more complex dynamics, including periodic, quasi-periodic and chaotic behavior, are observed (see Figure 3). Periodic and quasi-periodic behavior are associated with discrete power spectra with frequency peaks *η*_i_ that are commensurable (i.e., ∑*k*_i_ *η*_i_ = 0 for some non-zero integers *k*_i_) for the periodic state and incommensurable (i.e., ∑*k*_i_ *η*_i_≠0 for any set of non-zero integers *k*_i_) for the quasi-periodic case. Chaotic behavior is indicated by non-closed bounded trajectories in state space, broadband continuous spectra and positive Lyapunov exponents (see Figure 4). Here, chaotic regimes arise by traversing a homoclinic Shil'nikov bifurcation (see Figure 13 in [62]) for non-rational ratios between the frequencies of the stimulation and the intrinsic model kinetics. This route to chaos [77] has also been identified in more theoretical neural models (e.g., [78], [79]). Arnol'd tongues or mode-locking structures (i.e., entrainment regions in the parameter space [76]) are apparent in Figure 4 as a result of negative largest Lyapunov exponents. At low amplitudes, we observe several distinct ranges of such mode locking, which seem to merge or overlap at higher amplitudes. Note that chaotic “islands” occur at incommensurable ratios between stimulation frequencies and intrinsic limit cycles and interrupt frequency locking. For example, at a stimulus amplitude of *ζ* = 0.8, entrainment occurs for stimulus frequencies between 0<*η*≤0.06075, 0.06831≤*η*≤0.07403 and 0.09474≤*η*≤0.1206 interrupted by chaotic regimes between 0.004835≤*η*≤0.03464. At a stimulus amplitude of *ζ* = 2.4, entrainment occurs for stimulus frequencies between 0<*η*≤0.1447, 0.1545≤*η*≤0.1608 and *η*>0.1749 interrupted by chaotic regimes between 0.0365≤*η*≤0.04397, 0.05237≤*η*≤0.06818 and 0.08906≤*η*≤0.09547. Note that these entrainment ranges are rough estimates due to the finite sampling of the parameter space and due to the occurring “islands” of chaos. The chaotic regimes that are present in the parameter space feature a single positive largest Lyapunov exponent that is equal to the entropy of the attracting set (see Figure 4).

**Figure 5 pcbi-1002298-g005:**
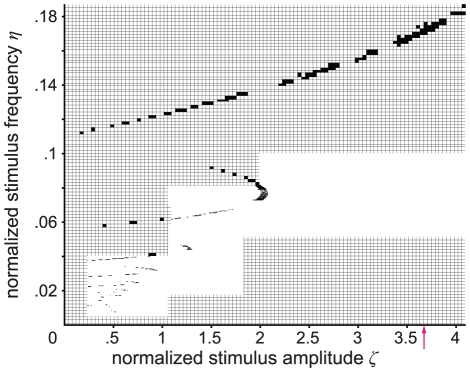
Occurrence of quasi-periodic behavior forming a two-torus surface in state space. Two-dimensional tori are indicated by two zero Lyapunov exponents (shown in black dots) in the parameter space of stimulus amplitude and frequency. The red arrow indicates the amplitude for which the experimental data fits best (see Figure 8 and Comparison section in Materials and Methods). The parameter regions of chaotic patterns that were selected for recomputing at a finer resolution (see Figure 4 and Figure 6) appear here mostly as white areas.

**Figure 6:**
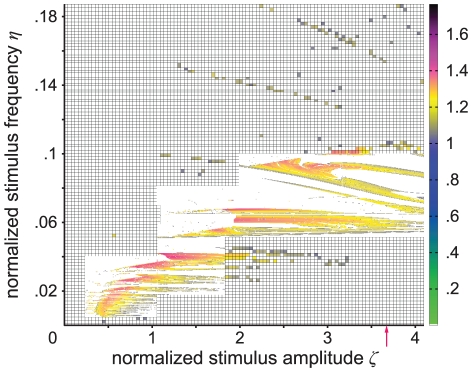
Kaplan-Yorke dimension of the periodically forced neural mass model in parameter space. The Kaplan-Yorke dimension *D*_KY_ given by Equations (8) and (9) never goes above 1.7, thus hyperchaos does not exist in the model. The red arrow indicates the amplitude for which the experimental data fits best (see Figure 8 and Comparison section in Materials and Methods). Several parameter regions with scattered, presumably fractal, patterns of chaotic regime were selected for recomputing at a finer resolution of normalized stimulus amplitude and frequency.

By studying the Lyapunov spectra, configurations are discovered where the system has two zero Lyapunov exponents and evolves on a two-dimensional invariant torus, indicating quasi- and bi-periodicity (see Figure 5). In general, the model is dissipative (i.e., the sum of Lyapunov exponents is negative) and does not exhibit hyperchaos, which is a higher form of chaos with at least two directions of hyperbolic instability on the attractor [80] (see Materials and Methods for further explanation), as seen from the observation that the second largest Lyapunov exponent is non-positive and the Kaplan-Yorke dimension (see Materials and Methods) never reaches or exceeds the value of two (see Figure 6). This means that the dynamics are low dimensional, not only for the periodic, but also for the chaotic regimes, as compared to the dimensionality of the system (which is six plus one dimension for the force). In general, the maximum Kaplan-Yorke dimension is a non-integer because of the complex geometry of the attractor. The periodic forcing seems to work mostly in the direction of entrainment, and although there are occasional “islands” of chaotic regimes, the regular forcing does not let the dynamics become exceedingly chaotic.

**Figure 7:**
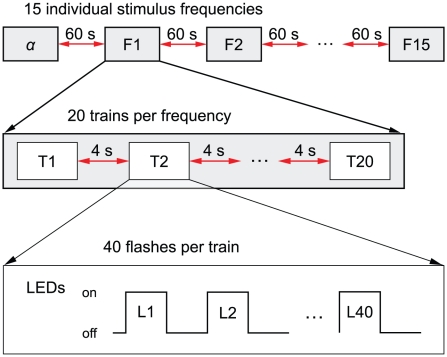
Experimental design of the flicker stimulation study. The LEDs were powered for half of each period. The raise and decay time for the LEDs was measured to be 100 µs.

**Figure 8:**
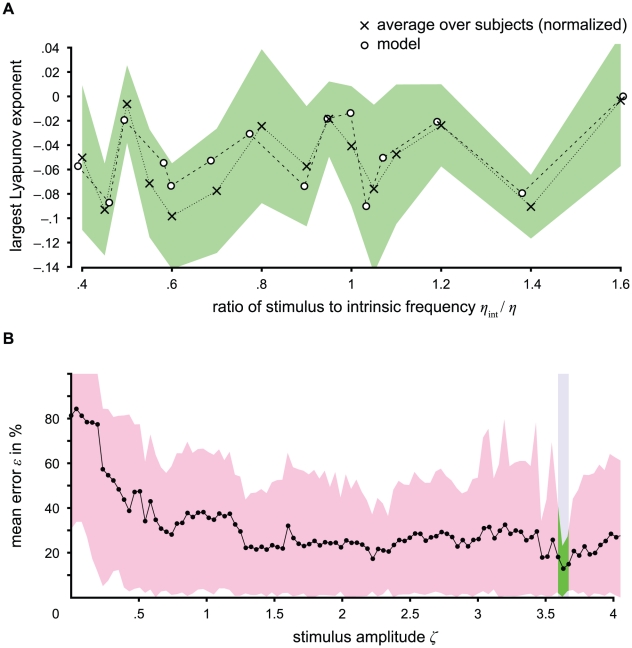
Comparison of model and data from a photic driving experiment. The largest normalized Lyapunov exponents calculated from the model show very good agreement with those obtained from experimental time series. The largest Lyapunov exponents for the average over all subjects and for the nearest neighbors of the model with the stimulus amplitude *ζ* that fits best (*ζ* = 3.6301) are plotted against the ratio of stimulus to intrinsic alpha frequency in (A). The largest Lyapunov exponents for the average over all subjects are normalized to the same range as for the model. The green area covers the standard deviation of the largest normalized Lyapunov exponents over all subjects. The comparison based on the minimization of the average of the minimum relative error (i.e., distance/maximum distance) between the normalized largest Lyapunov exponents of the model (comprising the four nearest neighbors) and of the experiment over stimulus frequencies is calculated for all stimulus amplitudes of the model. The mean error *ε* of model and average over subjects is shown in (B), where the red area covers the 5% and 95% quantiles. For the significant amplitude range, the quantiles are drawn in green. Significant amplitudes are consistent over subjects as shown in Table 1. For more details, see Comparison section in Materials and Methods.

**Figure 9:**
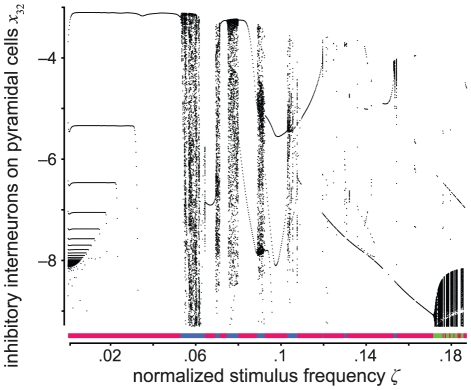
Bifurcation diagram for normalized stimulus amplitude *ζ* = 3.6301. The vertical axis is the normalized postsynaptic potential *x*_32_ on pyramidal cells caused by inhibitory interneurons, that is, the coordinate of the intersection points (black dots) of trajectories with the Poincaré hyperplane after neglecting initial transients. The horizontal axis is the normalized stimulus frequency. The regimes are color-coded and indicated by the horizontal line. Periodic regimes (red) exist, for instance, for frequencies ranging from 0 to 5.34·10^−2^. In this range, the system appears to undergo a period-adding bifurcation cascade by decreasing the normalized stimulus frequency. Chaotic (blue) and quasi-periodic regimes (green) occur, for example, for frequencies ranging from 5.3·10^−2^ to 6.23·10^−2^ (scattered dots) and between 17.21·10^−2^ and 18.72·10^−2^. The classification can be also taken from Table 2.

Furthermore, we find that the model is indeed able to explain frequency entrainment that is observable during a photic driving experiment (see also [1], [2], [81]). Note that Figure 5 in Schwab et al. [81] contains an error in the labeling of the *y*-axes. Each graph in this figure correctly plots the ratio of stimulus to response frequency (*y*-axis) against the ratio of stimulus to alpha frequency (*x*-axis). In this case, a horizontal line indicates an entrainment effect, while absence of entrainment would result in a diagonal line. We estimated the largest Lyapunov exponents from the data (see Methods section). In order to probe the stability of this estimate, we repeated it with the same data after adding various levels of Gaussian noise. The pattern of the Lyapunov exponents as function of stimulus frequency appears to be quite stable except for very low signal-to-noise-ratios SNR≤3 dB (see supplementary Figure S1). We compare our model outcome with these experimental Lyapunov exponents (see the section Experimental data in the Materials and Methods section and Figure 7 for the experimental paradigm) and find a particular stimulus amplitude for which, for all ten subjects, the model predicts Lyaponov exponents that are in close agreement with those estimated from the experimental data (Figure 8 and Table 1), with the amplitude being close to *ζ* = 3.6692 for all ten subjects. In seven of the subjects, the correlation between model prediction and measurement over stimulus frequencies was significant (*p*<0.05, corrected). A bootstrap test yielded a probability of error (significance) for the mean over subjects of 6.2% (see also Figure 8 (B) and Table 1). For three subjects (numbers 3, 6 and 7), the individual fit was not significant (see Table 1). Interestingly, this is reflected in the means and the standard deviations of the shift-and-scale parameters *u* and *v* (see the section Comparison in the Materials and Methods section and supplementary Figure S2). For the corresponding model configuration, we present a compact representation in Figure 9 and describe the system states qualitatively in Table 2. In the range of stimulus frequency *η* between 0 and 0.0534, the system performs limit cycles and appears to undergo a cascade of period-adding bifurcations [82] with descending stimulus frequency. This local bifurcation consists of saddle-node bifurcations in which a (*n*+1)-periodic orbit arises out of a *n*-periodic orbit for *n* ∈ N_1_ [83], [84]. For stimulus frequencies *η* above 0.0534, ranges of chaotic, periodic and quasi-periodic behavior occur. Due to the high dimensionality of the system, an instructive presentation in the form of a video is available, comprising orbits (PSPs), time series, and power spectra (see Video S1).

**Table 1:** Single subject comparison of largest Lyapunov exponents.

Participant	Normalized amplitude *ζ*	Mean errors *ε* in %	Significance (*p*<.05)
1	3.67	19	yes
2	3.67	22	yes
3	2.89	30	no
4	3.67	17	yes
5	3.67	16	yes
6	3.47	22	no
7	2.34	26	no
8	3.67	23	yes
9	3.67	20	yes
10	3.67	22	yes
Mean	3.6301	13	yes
Medial	3.6301	13	yes

Single subject comparisons fit the model at normalized stimulus amplitude around of approximately *ζ* = 3.67 with mean errors *ε* over all normalized stimulus amplitudes around 20%. The amplitudes that best fit the data are significant for the mean and median as well as for seven out of the ten subjects. The calculation is described in the Comparison section in Materials and Methods.

**Table 2:** Dynamic regimes occurring for normalized stimulus amplitude *ζ* = 3.6301.

Orbit	Limit cycle	Two-torus	Strange attractor
Occurrence in frequency range (·10^−2^)	>0 to 18.72	17.21 to 18.72	5.34 to 6.23; 7 to 7.18; 7.52 to 8.04; 8.92 to 9.29; 10.33 to 11; 11.96 to 17.21

A limit cycle appearing as periodic oscillations is a closed orbit in state space. An invariant torus indicates quasi-periodic oscillations that manifest themselves in the power spectra with peaks at incommensurate frequencies. A strange attractor is a bounded attracting set in which all trajectories are unstable and nearby trajectories locally diverge from each other exponentially, as evidenced by a positive Lyapunov exponent, as well as a broadband power spectrum.

## Discussion

In this study, we analyzed the behavior of the periodically forced extended Jansen and Rit neural mass model (NMM) as a function of amplitude and frequency of the stimulus within biologically plausible ranges. The system investigated exhibits interesting and complex dynamics, including chaos. As an important result, the model was able to account for the EEG dynamics of a photic driving experiment. Photic driving paradigms are of great importance in clinical practice [6], [7], [8]. In this type of experiment, the dominant brain rhythm during rest, the alpha rhythm (around 10 Hz), is entrained by a periodic visual stimulus.

### Relation to previous results

It should be pointed out that many aspects of our results are in close agreement with previous studies of other types of periodically driven oscillators (see, for example, [75], [76], [85]). Frequency entrainment effects have been described in, for example, the Rössler system [86], the Oregonator model [87] describing chemical oscillators such as the Belousov-Zhabotinsky reaction (e.g., [88]), the Duffing oscillator describing mechanical pendulums with flexible elements [89], [90], the van der Pol oscillator modeling electrical triode circuits [91], the Lorenz system describing turbulent convection in hydrodynamics [92], [93], and the Hodgkin-Huxley model of a neuron [94]. Overlapping or merging mode-locking regions in the parameter space were also discovered in a periodically driven van der Pol oscillator [95].

While reverse *periodic-adding* cascades appear to be the route to chaos in this study of a periodically forced Jansen and Rit model, in a number of previously investigated systems, cascades of period-doubling led to chaos, for example, in the Duffing oscillator [89], [90], [96], the Lorenz system [93], [97], the Rössler system [98], the Brusselator [99] and the Oregonator [87]. In the van der Pol oscillator, both routes – period-adding [83], [100] and period-doubling cascades – occur [91], [101]. On the other hand, our results concerning the route to chaos are in line with findings in a periodically stimulated excitable neural relaxation oscillator [27] and a simple model of the Belousov-Zhabotinsky reaction [102]. Crevier and Meister [103] describe retinal (*electroretinogram*, ERG) and cortical responses (LFP and *visual evoked potential*, VEP) to periodic flashes of light in salamander and humans. They also found complex behavior such as frequency entrainment in experimental data as well as in their model. In contrast to our findings, they found a cascade of period-doubling bifurcations (in both data and model) that leads to chaotic regimes in their model. Finally, quasi-periodic solutions have also been reported for various systems, such as the van der Pol oscillator [91], [101], the Oregonator [87], the Rössler system [98] and the Hodgkin-Huxley model [94], [104].

### Modeling the dynamics in photic driving data

We applied the concept of a periodically forced oscillator to model brain resonance effects. In the brain, such periodic input might stem from rhythmic stimulation of the brain, such as in the photic driving paradigm, or from the output of other oscillating brain areas. Such coupling between (oscillating) processes inside and outside the brain has been discussed as important for the processing of information (e.g., [105], [106], [107], [108], [109]). We described the dominant intrinsic brain rhythm using the NMM performing a self-sustained oscillation, generated by an Andronov-Hopf bifurcation [62]. Generally, resonance phenomena such as frequency entrainment in photic driving experiments can be explained by the concept of a periodically forced oscillator. Applying periodic input to an oscillatory system will change the current phase of the oscillation and frequency entrainment (i.e., phase locking) occurs if the sum of phase changes is nonzero over time [75] (see Results for more details). It is expected that the dynamics of the system depend on timing, that is, the ratio between stimulus and intrinsic frequencies, as well as the intensity of stimulation. While the general trend of the frequency-detuning curves is similar for our model and experimental data, there are numerous deviations (see Figure 2). These might be explained by the simplicity of the model. In the brain, many neuronal circuits are likely to be concurrently active and deviating behavior might be canceled out.

In our simulations, we found that the dynamics of the periodically forced extended Jansen and Rit NMM feature a rich mosaic of complex behavior (Figure 3). From the parameter space analysis presented in Figure 4, it can be seen that both flicker intensity and frequency are critical parameters. As expected based on theory [75], [76], the state space analysis reveals that the system is entrained by the stimulus frequency (see Figure 1) where the entrainment regions (i.e., plateaus in the frequency-detuning curve) around the intrinsic frequency become wider with increasing stimulus intensity (results not shown). Also, stimulus frequencies below the intrinsic frequency lead to decelerations of the intrinsic rhythm of the modeled cortex and vice versa. This phenomenon is reflected by the ratio of stimulus to response frequencies *η*/*η*_resp_ above and below the diagonal in Figure 1 for stimulus frequencies below or above the intrinsic frequency (i.e., *η*/*η*_int_<1 and *η*/*η*_int_>1), respectively.

In regions of the parameter space without entrainment, complicated interaction between stimulus and intrinsic kinetics leads to periodic, quasi-periodic, and chaotic behavior, as indexed by the largest Lyapunov exponents and the Kaplan-Yorke dimension. Areas with different dynamic behavior form fractal structures in parameter space (Figure 4 to [Fig pcbi-1002298-g005] Figure 6) so that rhythmic and chaotic brain states are found virtually next to each other and even small parameter changes can give rise to a switch from one to another. For these parameter configurations, different forms of the extrinsic periodic input would affect the specific pattern of chaotic regimes in the parameter space, but not the qualitative behavior if the parameter of the stimulus shape *δ* ranges between 109 and 130, as was found by additional simulations with different stimulus shape parameters (results not shown). On this account, the shape of the extrinsic input is an important model parameter for investigating the occurrence of complex regimes that needs to be investigated in the future.

It should, however, be pointed out that this result has been obtained from a purely deterministic model without any added or modulating noise. If noise is added to the input, this would cause jitter in its amplitude and frequency, and thereby impose a blur on the pattern depicted in Figure 4. However, the gross patterns are expected to survive; that is, areas with a high density of “chaotic” configurations (e.g., around 6 Hz and amplitudes between 4 and 5 mV) will feature a lower degree of predictability than areas without any configurations with positive largest Lyapunov exponents (e.g., around 11 Hz, same amplitude range). Strikingly, this is corroborated by the fact that the characteristic patterns of unpredictability generated by the model were also found with reasonable accuracy in the noisy experimental data (Figure 8). We identified a particular stimulus amplitude, where, for all subjects, the Lyapunov exponents are in close agreement between experiment and model (Figure 8 and Table 1). We found that the profile of the characteristic Lyapunov spectra for the stimulus amplitude that best fits the data is preserved when noise is added to the input for a signal-to-noise-ratio (SNR) up to 10 dB (for more details, see Model in the Materials and Methods section). The intensity that best fits our experimental data is located in the upper portion of the effective range for exciting inhibitory interneurons. Since the largest Lyapunov exponent reflects fundamental properties of the current dynamic regime of a system (as evidenced, for example, by its sensitivity to pathological states of the brain, such as epilepsy, see [72], [73], [74]), the fact that our model predicts its dependence on the most important stimulus parameter (frequency) corroborates the validity of the model.

Consequently, we predict that a decrease in stimulus intensity in photic driving experiments would shrink and an increase would broaden the ranges of frequency entrainment (i.e., the plateaus in the frequency-detuning curve). Our model also predicts that saturation effects become important starting with approximately 1.3 times the currently applied stimulus intensity and for intensities close to zero. A stimulus increase between 1 and 1.3 times the current intensity could lead to an improved entrainment effect (i.e., broadened range). Such broadening of the entrainment range is particularly important because in clinical practice, the individual alpha frequency is usually unknown. It is important to know how great an increase in the stimulus intensity still improves the entrainment effect and hence makes the photic driving more reliable.

Although the effect of photic driving has long been known, and standard examination in neurology includes intermittent photic stimulation in patients with suspected photosensitive epilepsy, the exact pathomechanism is not well understood. It is known that the *photoparoxysmal response* (PPR) is inheritable. In terms of electrophysiology, photosensitive epilepsy seems to be associated with changes in oscillatory activity. For example, Parra et al. [16] found enhanced gamma band synchrony and hypothesize that “ … some sort of recruitment or dynamic capture of neurons into larger assemblies appears to precede the epileptic chain reaction (ictal cascade) that ends in a paroxysmal oscillation, the PPR.” Likewise, Visani et al. [110] confirmed the potential importance of gamma band activity and found alpha band activity relevant to the PPR. Using transcranial magnetic stimulation, Siniatchkin et al. [111] found evidence that an increased excitability of the occipital but not the motor cortex might be associated with the PPR. The above studies indicate that a model including more than one area might be needed to further elucidate the pathomechanism of the PPR. Our model can be extended to give such experimental predictions or explanations for experimental findings. However, concrete simulations with, for instance, increased excitability in the occipital cortex and regular excitability in a second region are beyond the scope of this paper.

In short, we show that our model is capable of accounting for major aspects of the photic driving paradigm. This sets the scene for future work that will explore the predictions of the model in health and disease in more detail based on additional experimental data. Furthermore, a systematic exploration of the parameter space of the model with respect to brain resonance is needed. All this requires substantial efforts and is beyond the scope of the current proof-of-principle paper.

A principal limitation of our study is the modeling of the thalamus as independent signal generator, neglecting the cortico-thalamic feedback loop. However, we tested a model of the thalamus according to Robinson et al. [112] and found that, at least for the parameters of the cortical model used in this work, the simple signal generator approach yields a good approximation. Future work will include measurements and explicit modeling of the thalamo-cortical loops.

### Chaos in the brain?

Another issue which must be discussed is whether and to what extent our results support the idea of chaotic dynamics in the brain. The model investigated here describes complex, partially chaotic, dynamics at the mesoscopic spatial scale, which captures mass action of neural ensembles [28]. Chaotic dynamic regimes have been shown before in mesoscopic models of the cortex [113], [114] and of the olfactory bulb (e.g., [115]). Concerning the brain, there is evidence for chaotic behavior at different hierarchical levels, from single neurons to entire neural ensembles [116]. A suitable means to experimentally access neural activity at the mesoscopic level is provided by M/EEG, which records the summed activity of 10^5^ to 10^9^, mainly cortical, neurons [40], [41]. M/EEG data describe high-dimensional, noisy, nonlinear, and non-autonomous processes [117], which render it difficult to distinguish between stochastic and complex deterministic processes like deterministic chaos. Accordingly, although there is some evidence for chaos in such data (e.g., in epilepsy), the issue remains controversial (for a discussion, see [118] and the references cited therein). However, irrespective of whether the complexity of M/EEG fulfils the exact mathematical criteria of deterministic chaos, the parsimonious NMM, as shown here, helps to better describe the dynamics of such data and the underlying brain processes.

Apart from brain rhythms in characteristic frequency bands (e.g., the alpha rhythm), complex behavior with noise-like characteristics causes the continuous spectral components in these data. This can be interpreted as filtered noise (e.g., stochastic sensory input) or described by nonlinear deterministic processes. We have shown that periodically driven NMMs may explain the continuous spectral components of M/EEG without having to consider noisy input processes. Other NMM studies often apply a stochastic input process with the effect that the spectra are more realistically widened around an intrinsic frequency of interest (e.g., alpha band) (e.g., [23], [57], [58], [65]). It is, however, an advantage, if these continuous spectral components can be modeled and controlled as intrinsic phenomena of the neural circuits, because there is evidence that broad spectral components are also modulated by cognitive processes and hence their generative processes play a role in information processing (e.g., [119], [120], [121]). This is corroborated by the postulated prominent role of chaos in information processing (see, for example, [115], [116]).

Furthermore, the complex behavior of the NMM for certain parameter sets or ranges could be used to explain ordered sequences of dynamic regimes and multi-stability in M/EEG data by producing a temporal hierarchy [62]. Such ordered sequences have been observed in, for example, perception (e.g., mono- and binocular rivalry [122], Necker-cube illusion), stages of sleep [123], [124], changes in attention or vigilance, learning and training such as odor recognition [125], [126], progression of disease such as epilepsy [127], [128], [129], [130], and effects of medication. State transitions or multi-stabilities appear since the brain is subjected to multiple high-dimensional stimuli from both exogenous (e.g., vision or haptic) and endogenous processes (e.g., endocrine or circulatory system), and is highly dependent to the current on the current individual state (e.g., vigilance, sleep or attention). For example, one can interpret the quasi-periodic behavior in Figure 3 as multi-stability. However, the orbits are sensitive to noise, albeit in terms of fine structure and the associated sequences, rather than the overall structure.

One way to achieve ordered sequences of dynamic regimes that are sufficiently robust against noise is to adequately change the state space through parameter changes that are slower than the state dynamics producing a temporal hierarchy [62]. For example, one could incorporate a second model with kinetics slower than the NMM (e.g., representing metabolic processes or the neuroendocrine system) that controls a subset of the NMM parameters (e.g., couplings in terms of synaptic plasticity). In this way, the dynamic behavior of the NMM may change qualitatively through passing bifurcations and thus sequences of the complex regimes will be occur. A fine example of this approach is provided by Steyn-Ross et al. [131], who modeled the succession of slow wave and REM sleep phases in humans using a mean field model. The parameters of the model were controlled by the states of a low-kinetics model describing the levels of acetylcholine and somnogens (such as adenosine).

Note that the directions of parameter changes play an important role in parameter ranges of the system where a hysteresis occurs (see, for example, in [62] Figure 2 and Figures 4 to [Fig pcbi-1002298-g005] 6: branch type-I A and B, and type-II AB to CC). This previous study [62] provides a catalogue of regimes that is potentially helpful to prevent the system from hysteretic behavior or, quite the reverse, to perform hysteresis.

The present study is the first to find complex types of behavior like entrainment, chaos, and periodic and quasi-periodic motion in a periodically forced Jansen and Rit NMM for a single cortical area for biologically plausible parameter ranges without considering noise processes. Such dynamics are observable in brain data and relevant to the explanation of brain function. We demonstrate that with the NMM, one can explain brain resonance phenomena like frequency entrainment in a clinically relevant photic driving experiment. It should be pointed out that, at this stage, the aim of our model has not been to directly improve the diagnostics of mental illnesses, but rather to allow deeper understanding of the mechanisms underlying a diagnostic tool and thereby pave the way for future new treatments and diagnosis techniques. As a logical next step, the model should be applied to pathological cases in order to specify what disease-specific inferences can be made.

### Simplifying assumptions in the model

As any model, our model features a number of simplifications with respect to reality. The mean-field model studied embodies structural (e.g., local neural circuitry) as well as functional approximations (e.g., mean postsynaptic potential (PSP), mean firing rates and its conversions) of neural circuits to describe brain dynamics at the mesoscopic and the macroscopic levels, which are accessible, for instance, to LFP and M/EEG. Simplifications appear at all levels of modeling: the description of single cell behaviors, the modeling of neural masses (NMs) based on a single cell description (i.e., firing rate neuron), the description of the local neural circuitry and the description of networks of brain areas.

On the single cell level, we consider the firing rate instead of individual action potentials. Moreover, only two types of synaptic kinetics are modeled, which leads to two types of neurons that either excite or inhibit other neurons. In the brain, there is a great diversity of electrophysiological neuron types that differ in their specific input and output operations [43].

On the population level, the distribution of states (i.e., PSPs and firing rates) is described by their means, while variances and higher-order statistics are left out of consideration.

The local neural circuitry of the cortex is characterized by a wealth of distinguishable populations and their interconnections (see, for example, [55]). In the model structure used here, this is simplified by simply considering pyramidal cells and two feedback loops established by inhibitory and excitatory interneurons.

Finally, in this work, we deal with a cortical area mean-field model, without considering projections to the rest of the brain. In particular, the thalamus, which might play a role here, is modeled only in terms of its output.

Moreover, since the retina is fully illuminated by the flicker that drives much of the visual cortex (see Materials and Methods for further explanation), we consider the entire primary visual cortex as a single source using a simple NMM for a single cortical area. Of course, the primary visual cortex is much more complicated than a single Jansen and Rit circuit, not to speak of its incorporation in brain-wide networks. Hence, our model can only represent a subset of the dynamics of the entire system. Nonetheless, our results show that the model can account for the main phenomena in the photic driving paradigm. However, one must be aware that the future availability of new or more detailed data might necessitate an extension of the model.

### Link to extended brain networks

In this study, we show that a simple local cortical area model is already capable of performing relevant complex dynamics, particularly in response to periodic inhibitory feed-forward stimulations. Based on the fact that such a local cortical circuitry of neural populations is embedded in large-scale networks that can span the whole brain and also include subcortical structures such as the thalamus, the question arises to what extent network interactions might contribute to the complexity of brain signals such as M/EEG.

The present work might also contribute to the understanding of large-scale networks. In particular, the present results can be applied to inhibitory feed-forward interactions in networks between two local area models, where one model periodically performs spikes that drive the other model. The frequency entrainment or locking phenomena we found here can thus be interpreted as an effect of network interactions, which might have an impact on functional or effective brain connectivity measures such as the phase correlation (e.g., [109], [132]) or the Granger causality (e.g., [133], [134]).

From the modeling perspective, one can obtain a dynamic regime of a local cortical area such as quasi-periodic behavior within a network by frequency locking through feed-forward inhibition from another local area by considering the following steps: (i) tuning the driving cortical area so that it performs (spiky) rhythms, (ii) selecting the dynamic regime depending on stimulus amplitude and frequency (see Figure 4 to [Fig pcbi-1002298-g005] Figure 6), (iii) adjusting the characteristic time constant of the driver to tune the frequency of the driver to the required stimulus frequency, and (iv) selecting the characteristic potential and/or the coupling parameter so that amplitude of the driver fits. The parameters for the driving cortical area can be taken from the catalogue of regimes presented in our previous work [62]. Moreover, in order to best reproduce a specific phenomenon, for instance in M/EEG data, this catalogue helps to balance the complexity of a network, in particular, whether a single area model is sufficient or not. The effective extrinsic input ranges of a cortical area model [62] can be used to determine the coupling parameters between areas in order to prevent a network or individual cortical areas from saturating.

The use of these approaches to control or set up a network depends on the complexity of the graph. For instance, several bidirectional connections or feedback loops usually make a setting more difficult. In such complex graphs, one can expect more complicated behavior than for a single local area model, such as hyperchaos or phase locking of several (chaotic) regimes. However, one might to have to perform a separate analysis for the network.

## Materials and Methods

### Model

A generative model for brain measurements such as M/EEG can be specified by two separate systems: the state system *f* explaining the usually hidden neuronal states **x** (e.g., the mean postsynaptic potentials (PSPs) of neuronal populations that potentially generate M/EEG), and the observer system *g* relating the neuronal states to the measurements **z**:
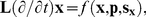
(1)and
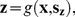
(2)where **L**(*∂*/*∂t*) is a temporal differentiation operator, **p** denotes the extrinsic inputs, and **s_x_** and **s_z_** parameterize state and observer system, respectively. For the state system *f*, we use a neural mass model (NMM) of a cortical area. For the observer system *g*, we use a simple linear relationship, as we simply consider one area (i.e., source), because the retina is fully illuminated during the photic-driving experiment that presumably drives much of primary visual cortex in parallel, and therefore no elaborate forward modeling is needed. The state system will be explained in more detail in the following paragraphs.

The NMM of Jansen and Rit [23], [52] describes a local network representing a cortical area. This basic circuit, consisting of three interacting neural masses (NMs), namely *pyramidal cells* (PCs: NM 3) with feedback loops mediated by *excitatory* and *inhibitory interneurons* (EINs and IINs: NMs 1 and 2), has been described in a number of previous studies (e.g., [23], [44], [52], [53], [57], [58], [59]).

Note that the feedback loops may also be modeled dynamically (see, for example, [26], [48], [112] when also considering propagation delays, see, for example, [135], [136], [137]). However, here we assume connections within a single area, resulting in transmission times which are shorter than the characteristic (dendritic) time constant *τ* = 10 ms. Therefore, it is sufficient to describe the feedback connection by a gain constant.

With this NMM, the mean neuronal states can be described by a system of six nonlinearly coupled first-order ordinary differential equations:

pyramidal cells (3) to (excitatory and inhibitory) interneurons (1 and 2, combined to 0)
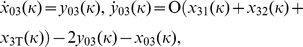
(3)
excitatory interneurons (1) to pyramidal cells (3)
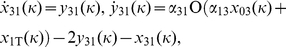
(4)
inhibitory interneurons (2) to pyramidal cells (3)
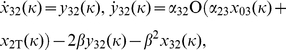
(5)

where the state vector **x** = (*x*_03_, *x*_31_, *x*_32_, *y*_30_, *y*_31_, *y*_32_)^T^ contains the normalized mean PSPs *x_ba_* and currents *y_ba_* at NM *b* caused by NM *a*. The extrinsic afferents T projected to NM *b* are denoted by *x_b_*_T_. The average synaptic gains or the average numbers of synaptic contacts established between the two NMs *a* and *b* are represented by the constants *α_ba_*. Furthermore, *β* is the ratio of *e*xcitatory to *i*nhibitory dendritic time constant *β* = *τ*_e_/*τ*_i_ and, in the formulas (3) to (5), the dot indicates the derivatives with respect to the normalized time *κ* = *t*/*τ*, where *τ* is the characteristic time scale. The transfer function O(*x_b_*) that converts the normalized mean PSP *x_b_* = Σ*_a_ x_ba_* (i.e., the normalized potential at the axonal hillock) to the normalized mean firing rate is taken to have a sigmoidal shape O(*x_b_*) = 1/(1+*γ* exp(−*x_b_*)), where *γ* represents the distribution of firing thresholds within a NM. The normalized and generalized Equations (3) to (5) correspond to the Jansen and Rit model [23] with the characteristic time constant *τ* = *τ*_e_, the coupling parameter *α_ba_* = 2*e*_0_ *r c_ba_ H*_e,i_ *τ* ^2^/*τ*_e,i_, the sigmoid parameter *γ* = exp(*υ*_0_ *r*), and the states *x_ba_*(*κ*) = *r_b_υ_ba_*(*τκ*), with the following parameters: maximum firing rate 2*e*_0_, the slope of the sigmoid *r*, the mean number of synaptic contacts *c_ba_*, and the *e*xcitatory and *i*nhibitory synaptic gains *H*_e,i_ (for more details, see [23], [62]). Note that we use normalized parameters and variables in the rest of this paper without further indicating this.

In this work, we explore the dynamics of the single-area model as a function of amplitude and frequency of a periodic input. This input consist of brief pulses similar to ones used by Jansen and Rit for eliciting visual evoked potentials [23], [52], or used in dynamic causal modeling (e.g., [63], [66]). These pulses are meant to represent the impulse response of the visual pathway, which has been investigated experimentally by a number of researchers (see [52], and the references cited therein) and described analytically by Watson and Nachmias [138].

In the following, we will specify the parameter space to be investigated. The system described by Equations (3) to (5) has nine parameters, namely couplings *α_ba_* with *ba* = {13, 23, 31, 32}, kinetic ratio *β*, sigmoid parameter *γ*, and extrinsic inputs *x_b_*_T_ with *b* = {1, 2, 3}.

Jansen and Rit [23] proposed a specific parameter set for the NMM of a cortical area, based on a thorough discussion of the literature. The normalization of time and potentials by Jansen's excitatory dendritic time constant and sigmoid slope (*τ* = 10 ms and *r* = 0.56 mV^−1^, respectively) leads to the following dimensionless parameters in our model: couplings *α*_13_ = 12.285, *α*_23_ = *α*_13_/4, *α*_31_ = 4*α*_13_/5, *α*_32_ = −11*α*_13_/13, kinetic ratio *β* = 0.5 and sigmoid parameter *γ* = 28.7892. The extrinsic inputs on the three NMs are taken to be constant for EINs *x*_1T_ = 0 and PCs *x*_3T_ = 3.36, and time-variant for IINs in the form of periodic pulses *x*_2T_ = *ζ* exp(−2*δ* cos^2^(*θ*)), with the angle *θ*

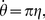
(6)specified by stimulus amplitude *ζ* and stimulus frequency *η* (*δ* controls the shape and is set to *δ* = 110). Such a peaky waveform has been found in the lateral geniculate nucleus of the thalamus in response to square visual stimuli [139]. Interestingly, a very similar waveform can be generated using a NMM of the thalamus, as proposed by Robinson et al. [112], which takes into account the intra-thalamic and thalamo-cortical feedback loops (e.g.,[140]). In this model, a strong inhibitory influence of the reticular nucleus on the thalamic relay cells during the relaying of external sensory stimulation, such as an on/off waveform of flickering lights, sharpens the cortical input to render it pulse-like. The time-variant input to the IINs may represent thalamic feed-forward input. This type of disynaptic *feed-forward inhibition* has been described as crucial for bottom-up processing in the somatosensory (e.g., [141], [142]), auditory (e.g., [143]), and visual (e.g., [144], [145]) systems of rodents. Moreover, the literature provides evidence that feed-forward inhibition (e.g., from layer IV IINs driven by thalamus) dominates excitation (from thalamus) (e.g., [144], [146]). Also, our previous model analysis of the Jansen and Rit circuit reveals the importance of input on IINs for controlling cortical behavior [62].

In the absence of stimulation (i.e., *x*_2T_ = 0), the system intrinsically performs limit cycle oscillations arising from Andronov-Hopf bifurcations, appearing as harmonic oscillations with a frequency of approximately *η*_intr_ = 0.108 (see bifurcation diagram and phase portraits, Figure 2 and Figure 3 in [62]). Applying the characteristic dendritic time constant *τ* = 10 ms as defined above, this corresponds to the parameter set proposed by Jansen and Rit [23] and an actual oscillation frequency of *f* = 10.8 Hz, and can be used to describe alpha rhythms in brain signals. This characteristic time constant is used in all results reported in this work. Note, however, that varying the characteristic time constant *τ* only scales the neuronal states **x**(*κ*) in time *t* = *τ κ* and thus the frequency *f* = *τ* ^−1^ *η* while the states **x**, the form of time signals and the underlying mechanisms such as bifurcations remain unaffected. Hence, the frequency depends on the choice of the characteristic time constant *τ* and thus the normalization embraces all cases of *τ*. In order to study the system with periodic stimulation around the intrinsic frequency (*η*_intr_ = 0.108), the stimulus frequency *η* is taken to range from 0 to 0.19. The stimulus frequency is nonlinearly sampled ensuring (*η* Δ*κ*)^−1^∈N_1_ with the sampling interval Δ*κ*, so that the pulses are well sampled. The stimulus amplitude *ζ* is linearly sampled from 0 to 4.1 to cover the effective range of excitatory inputs on IINs within the limit cycle which exists when extrinsic input on IINs is constant (see Figure 8 in [62]). Since the specification of the effective extrinsic input ranges is based on an analysis of the invariant transfer function (sigmoid function) [62], these ranges are valid for any type of input, no matter whether it is constant or time-variant.

In summary, for analysis, we consider a system of seven first-order ordinary differential equations (Equations (3) to (6)) describing the (neuronal) states x^*^ = (*x*_03_, *x*_31_, *x*_32_, *y*_30_, *y*_31_, *y*_32_, *θ*)^T^ specified by two parameters **p** = (*ζ*, *η*)^T^.

We study the differential equations (3) to (6) numerically using the fourth-fifth order Runge-Kutta method over *κ* = 30**·**10^3^ in time (which equals 5 minutes for *τ*_JR_ = 10 ms, according to Jansen and Rit [23]) with a relative tolerance of 10^−11^, and then linearly sampled with an interval Δ*κ* = 10^−2^ for further analysis. From the last 6·10^3^ samples (last minute if *τ*_JR_ = 10 ms), the histograms of each state were computed using the optimal number of bins [147]. Using the state equations, we also compute the characteristic mean frequency of each attractor [85]. Characteristic mean frequency is the time average of a trajectory over the angle velocity at points along an *n*-dimensional curvature forming an attractor in state space. To study the complex behavior, we computed the power density spectra of the time series (last 6·10^3^ samples) using the fast Fourier transform, especially for the time series of the PSPs of the PCs, which are reflected in M/EEG.

We also compute the characteristic Lyapunov spectra, that is, all six Lyapunov exponents *λ*_1_>*λ*_2_ …>*λ*_6_ directly from the differential equations (3) to (6), using the Fortran algorithm by Chen et al. [148], integrated for *κ* = 1073,742.00 using a constant sample interval Δ*κ* = 10^−3^. Chen et al. [148] used a constant time-step fourth-order Adams-Bashforth integration method and a QR-reorthoginalization that also preserves orthogonality for higher-dimensional systems. The time interval is sufficiently long to stably estimate the characteristic Lyapunov spectra (error<10^−6^). The Lyapunov spectrum gives a quantitative measure of the sensitivity of the states of the system dependent on the initial conditions, or, more precisely, the average rate of divergence or convergence of two neighboring trajectories in the state space. Furthermore, the whole Lyapunov spectrum enables statements of hyperchaos.

Hyperchaos is a higher form of chaos with at least two rather than one directions of hyperbolic instability on the attractor [80] indicated by two or more positive Lyapunov exponents and by a Kaplan-Yorke dimension larger than two. Such a hyperchaotic attractor appears as a ‘folded-towel’ structure through a continuous stretching and folding in, at least, two independent directions of the state space [149]. Such behavior was first reported by Rössler in 1979 [80]. Generally, a system that performs hyperchaos must be of at least four dimensions.

Due to the computational effort required, only the largest exponent or a few of the largest ones are calculated in most of the existing literature. Here, we compute the whole characteristic Lyapunov spectra running on a massive parallel cluster system of the advanced computing unit at the Computer Center, Ilmenau University of Technology. We also select several regions from the parameter space with scattered, presumably fractal, patterns of chaotic regimes (i.e., positive largest Lyapunov exponents) for recomputing at a finer stimulus amplitude and frequency resolution (see Figure 4 through [Fig pcbi-1002298-g005] Figure 6).

We probe the stability of the characteristic Lyapunov spectra by adding a stochastic term to the stimulus *x*_2T_. Although the Gaussian noise process that we used is not autocorrelated and could lead to errors due to the constant integration step size of the Adam-Bashforth method, the estimation of the characteristic Lyapunov spectra (for the stimulus amplitude that fits the experimental data best) is stable up to a signal-to-noise-ratio (SNR) of 10 dB, especially for the 1∶1 entrainment region (i.e., *η*≈*η*_int_). However, the stochastic term changes the characteristic Lyapunov spectra specifically, for instance, at stimulus frequencies *η* around 2/3 of the intrinsic frequency *η*_int_. For this stimulus frequency range (0.5817<*η*/*η*_int_<0.7632), the profile is qualitatively preserved for mild noise with a SNR up to 17 dB. We determined the SNR as the ratio of the variances of the deterministic and stochastic portions of the stimulus. The variance of the deterministic terms *σ* ^2^(*x*_2T_) (i.e., periodic pulses) is given as follows
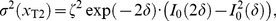
(7)where *I*_0_ is the modified Bessel function of the first kind, *δ* is the shape parameter and *ζ* is the amplitude of the stimulus *x*_T2_.

The knowledge of the whole spectrum enables us to derive the Kaplan-Yorke dimension [150] given by
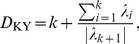
(8)where *k* is such that
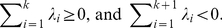
(9)


The Kaplan-Yorke dimension measures the upper bound of the Hausdorff dimension and is similar to the information dimension (entropy) or correlation dimension of an attractor. The Hausdorff dimension quantifies the complexity of the geometry of the attractor. For example, the Hausdorff dimension of a point is zero, of a line is one, of a plane is two, but irregular sets, such as fractals or the attractors found in this work, can feature non-integer Hausdorff dimensions. We divide the state space by classifying the behavior of the system qualitatively. To that end, we specify a Poincaré map *P* by choosing a suitable hyperplane transverse to the limit cycle of the unperturbed system. A Poincaré map *P* considers the intersections of a trajectory existing in the *d*-dimensional state space with a hyperplane of dimension *d*−1. The resulting discrete series of intersection points allow the characterization of the dynamics near periodic solutions. Finally, to study the relationship between system perturbation and system response in terms of synchronization and frequency entrainment, we compute the frequency-detuning curves [76]; that is, the difference of the response frequency (characteristic frequency or largest peak in the spectrum) and the stimulus frequency plotted against the stimulus frequency.

### Experimental data

Experimental data were obtained by performing a photic driving experiment that was adapted to the individual alpha frequency of the subjects. Data were previously published by Schwab et al. [81]. The aim of this former study was the quantification of frequency entrainment in the alpha rhythms that was most effective in the region around individual alpha and half alpha. Ten healthy participants (22 to 40 years of age, 5 male; 5 female) were stimulated by an intermittent flickering light, while EEG (32 channels, enhanced 10-20 system with a 10-10 system over the occipital region, Compumedics Neuroscan, El Paso, USA) was recorded. EEG was sampled at 1000 Hz and hardware-filtered between 0.1 Hz and 300 Hz. An initial resting condition of 60 seconds was recorded to define the individual alpha rhythm of each participant. The individual alpha frequency measured ranged from 9.5 Hz to 11.8 Hz. After this period, flicker stimulations were conducted for 15 fixed frequencies with an alpha ratio (stimulus/individual alpha frequency) ranging from 0.4 to 1.6 in each participant (randomized order of presentation). The flicker stimuli were generated by two LEDs outside the measurement chamber and were delivered via optical fibers to about 9 cm in front of the closed eyes of the subjects in order to ensure relatively stable luminance over subjects and a fully illuminated retina. The closed eyelid diffuses the flickering light from the optical fiber (with its viewing angle) so that the whole retina is illuminated (e.g., [151], [152]). Each stimulation frequency was presented in a sequence of 20 trains. A single train contained 40 flashes and was followed by a resting period (4 s). The complete experimental design is summarized in Figure 7.

One EEG channel located in the occipital region (O_1_) was examined per participant. Data were filtered and down-sampled to 200 Hz. For each participant, periods of 62.5 s (*n* = 12500 data points) were analyzed for the 15 flicker frequencies presented (the shortest available data length of the individual flicker blocks F1 through F15 is 62.5 s over all participants investigated).

The estimation of the largest Lyapunov exponent was based on the approach of Wolf et al. [153]. An embedding dimension of 16, a time delay of 9 (≈50 ms) and an evolving time of 5 (≈25 ms) was used for the investigation of flicker stimulations. Embedding parameters were defined according to Atay and Altintas [154].

### Comparison

Our periodically driven deterministic model exhibits chaos or otherwise complex behavior in certain parameter ranges (see Figure 4). A chaotic regime can be considered as a source of noise. Empirical data as from the photic driving experiment (see Experimental data) generally represent a highly noisy (nonlinear) signal. The sources of this noise are diverse and range from technical noise (e.g., Johnson-Nyquist noise of sensors, 50/60 Hz powerline interferences), via non-brain biological noise (such as transpiration, muscle activities of the heart or eye movements), and unrelated brain activity, to the chaotic behavior of the actual stimulus processing. Thus, mapping experimental data to any biologically motivated model with reasonable accuracy is an extremely challenging task. In particular, comparing identified system variables from model and data with respect to their absolute values needs to be done delicately. Consequently, we chose to compare the Lyapunov exponents as scalar measures of the variations of the regimes of the system with respect to a well-defined “external” parameter, here the stimulus frequency (available for both model and experiment). In contrast to the experimental design, our model has the stimulus amplitude *ζ* as a parameter in addition to the stimulus frequency *η*. For this reason, we searched for the stimulus amplitude where the model best fits the data.

The calculated 15 largest Lyapunov exponents from our experimental data are all positive due to the background noise. For our model, this is the case only if chaos arises (see Figure 4). The absolute values of the Lyaponov exponents can therefore not be compared directly. If, however, we assume that the unpredictability of the experimental data is partially due to background noise (which does not depend on the stimulus frequency) and partially due to the intrinsic dynamics of the modeled system, it makes sense to compare the pattern of dependency of the Lyapunov exponent from the stimulus frequency instead. For this reason, we compared the largest Lyapunov exponents as computed from the model to the largest Lyapunov exponent computed from the data, normalized to the same range as the model-based exponent (−9.6972·10^−2^≤*λ*_1, Model_≤−9.4435·10^−5^), by a shift-and-scale transformation *u*+*v*·*λ*_1_. For the means and standard deviations of *u* and *v*, please refer to supplementary Figure S2. Interestingly, the three subjects for whom the individual fits were not significant (number 3, 6 and 7; see Results section) are clearly noticeable here in terms of means and standard deviations of *u* and *v*. The offset of the experimental Lyaponov exponents *u* can be regarded as a multiplicative process *R* of divergence *S*_1_(*κ*), because |*S*_1_(*κ*)|≈*R*_1_•exp(*v κ·λ*_1_) with *R*_1_ = exp(*u*). Background activity and more general unspecified processes may be included in *R*.

Since the ratio between the sampling rates on the frequency axis is 4.6 between the model (69 samples) and the experimental data (15 samples), we compare an experimental data point with the four nearest neighbors in the model. The comparison and detection of the model configuration that fits the experimental data best comprise seven steps: (i) select the four nearest neighbors along the stimulus frequency axis of a amplitude configuration of our model to a query point in the experiment (i.e., the response to an experimentally applied ratio of stimulus to intrinsic alpha frequency), (ii) calculate the Euclidean distances in the plane spanned by frequency and largest normalized Lyapunov exponent between each of the nearest neighbors and the experimental data point (this way an agreement between model and data Lyaponov exponents is weighted according to the agreement between the frequencies they belong to), (iii) determine the maximum Euclidean distance between the nearest neighbors and the experimental data point, where the largest normalized Lyapunov exponent of a nearest neighbor is set to the lower (min(*λ*_1, Model_) = −9.6972·10^−2^) or to the upper bound (max(*λ*_1, Model_) = −9.4435·10^−5^) of the model-based largest Lyapunov exponent if the query point in the experiment is greater than −4.8533·10^−2^ (i.e., min(*λ*_1, Model_)/2+max(*λ*_1, Model_)/2) or not, respectively, (iv) calculate the relative error as the ratio of the distance of a nearest neighbor to its maximum distance, (v) detect the nearest neighbor with the minimum relative error for each experimental data point and average these errors over all 15 data points (for the different experimental frequencies), (vi) repeat steps (i) to (iv) for each amplitude of the model stimulation, and (vii) find the model configuration with the stimulus amplitude that fits the data best by detecting the minimum of the averaged minimum relative errors (i.e., mean error *ε* in graph B of Figure 8).

In order to test the significance of the comparison results, we (i) compute Pearson's (linear) correlation coefficient for each stimulus amplitude *ζ* as well as for each subject (and also for the average over subjects) between the largest normalized Lyapunov exponents of model and data as function of the ratios of stimulus to intrinsic alpha frequency, and (ii) test its significance by applying a Student's t-test. Due to the multiple comparisons of the 106 amplitudes and each four nearest neighbors, we used the Bonferroni correction for the significance level *p* = 0.05 corrected by *p*′ = *p*/(4*·*106).

Moreover, in order to further substantiate our findings, we performed a bootstrap test. We randomized the sequence of experimental Lyapunov exponents along the frequency axis so that their distribution remained the same but any putative frequency-dependence was destroyed. Then we applied our fit method and recorded the fit error. We repeated this 5000 times and obtained an estimate of the error distribution. Counting the occurrences of errors that are below the one obtained with the true sequence of frequencies, we obtained an estimate of the probability that such an error could have been achieved by chance.

## Supporting Information

Figure S1 **Largest Lyapunov exponents of the empirical data under the presence of noise.** The exponents are characterized by mean (white lines) and standard deviation (red area) over subjects with: **A** no noise and noise; **B** SNR = 13 dB; **C** SNR = 10 dB; **D** SNR = 7 dB; **E** SNR = 3 dB; and **F** SNR = 0 dB.
(EPS)

Figure S2 **Means and standard deviations of shifting and scaling parameters** ***u*** **and** ***v*** **for all ten subjects.**
(EPS)

Video S1 **Impact of stimulus frequency for normalized stimulus amplitude** ***ζ*** **= 3.6301.** The large diagram in the middle shows the attractor as a function of the postsynaptic potentials of the three neural masses: the normalized potential that inhibitory interneurons (IINs) cause on pyramidal cells (PCs), the normalized potential that excitatory interneurons (EINs) cause on PCs, and the normalized potential that PCs cause on both interneurons (INs). The small diagrams in the upper left- and right-hand corner show the corresponding time series and the spectra (amplitude and time is normalized) respectively. The heading provides information about the current normalized stimulus frequency that increases with time. The video shows the unperturbed system for the first six seconds with the limit cycle (large diagram) producing a sinusoidal rhythm (left diagram) that appears as a narrow peak at *η* = 0.108 in the spectrum (right diagram). All three curves (all in red) remain visible for the rest of the video for comparison. The three curves corresponding to a current perturbation are shown in green.
(AVI)
